# 

**Published:** 1908-03-15

**Authors:** 


### ANNOUNCEMENTS.

MISSOURI STATE DENTAL ASSOCIATION. The 43rd Annual Meeting of the Missouri State Dental Association will convene in St. Louis, June 1, 2, 3, 1908, at the Planters Hotel. Rates $1.50 and up per day. Efforts are being made to make this the most successful meeting in the history of the Association. Distinguished members of the profession from out of the State will be present. All ethical members of the profession are cordially invited to come.

E. P. Dameron, Secy.

THE NORTHERN OHIO DENTAL ASSOCIATION. The fifty-first annual meeting of the Northern Ohio Dental Association will be held at Canton, Ohio, May 26, 27, 28, 1908.

The sessions will be held in the City's Auditorium, one of the largest in the middle west, with headquarters at the Courtland Hotel. There are numerous other hotels in Canton so there will be accommodations for all. Hotel rates may be had at from $1.50 to $5.00 per day, American plan.

Canton is essentially a dental manufacturing town, having three large and busy plants. The exhibits will be first class.

The committees are sparing no time or expense to make this an especially attractive meeting. The program will be up to the standard of previous years. Men of international reputation have been secured to read papers and clinic.

Remember the time and place, May 26, 27, 28, 1908, Canton, Ohio.

The Executive Committee,
W. H. Whitslar,
J. H. Wible,
F. M. Casto, Chairman.

---

ST. LOUIS DENTAL COLLEGE CLINIC. The Alumni Association of the St. Louis Dental College wish to announce that their Annual Clinic will be held at the College building (Grand and Caroline St.) Tuesday and Wednesday, May 19th and 20th, 1908.

A good program is being arranged and all graduates of the College are repectfully requested to be present and aid in making this meeting a success.

Yours truly,

W. M. Collins }

T. F. Fleming } Committee.

THE WEST VIRGINIA BOARD OF EXAMINERS. The next regular meeting of the West Virginia State Board of Dental Examiners will be held at Huntington June 10, 11, and 12.

All applications together with the fee of $25. 00 should be sent to the Secretary not later than June 5th.

Blanks and other information can be obtained from

J. F. Butler, Secy.,
Charleston, W. Va.

---

MICHIGAN STATE DENTAL SOCIETY, ANNUAL MEETING AND BOAT-TRIP COMBINED.—The Michigan State Dental Society will hold its annual meeting on Wednesday, Thursday, Friday and Saturday, June 10th to 13th, inclusive, on board the steamer City of Mackinaw, on a trip through the Detroit River, Lake St. Clair, The Flats, and on to Mackinaw and the "Soo." The total expense of the trip, including passage, meals and berth, will be nineteen dollars for the round trip, from Detroit to Soo and return, and all our ethical dentists and friends are cordially invited to join us.

The principal feature of the meeting will be table clinics, good papers, a complete dental exhibit and a good time. Four days to find out what your fellow-practitioners are doing, an ideal meeting under ideal conditions.

Those desiring to have accomodations reserved for them should apply at once to Doctor O. W. White, 406 Fine Arts Building, Detroit, stating the number of persons in party, and whether it is family party or all men. A deposit of five dollars is required for each reservation.

